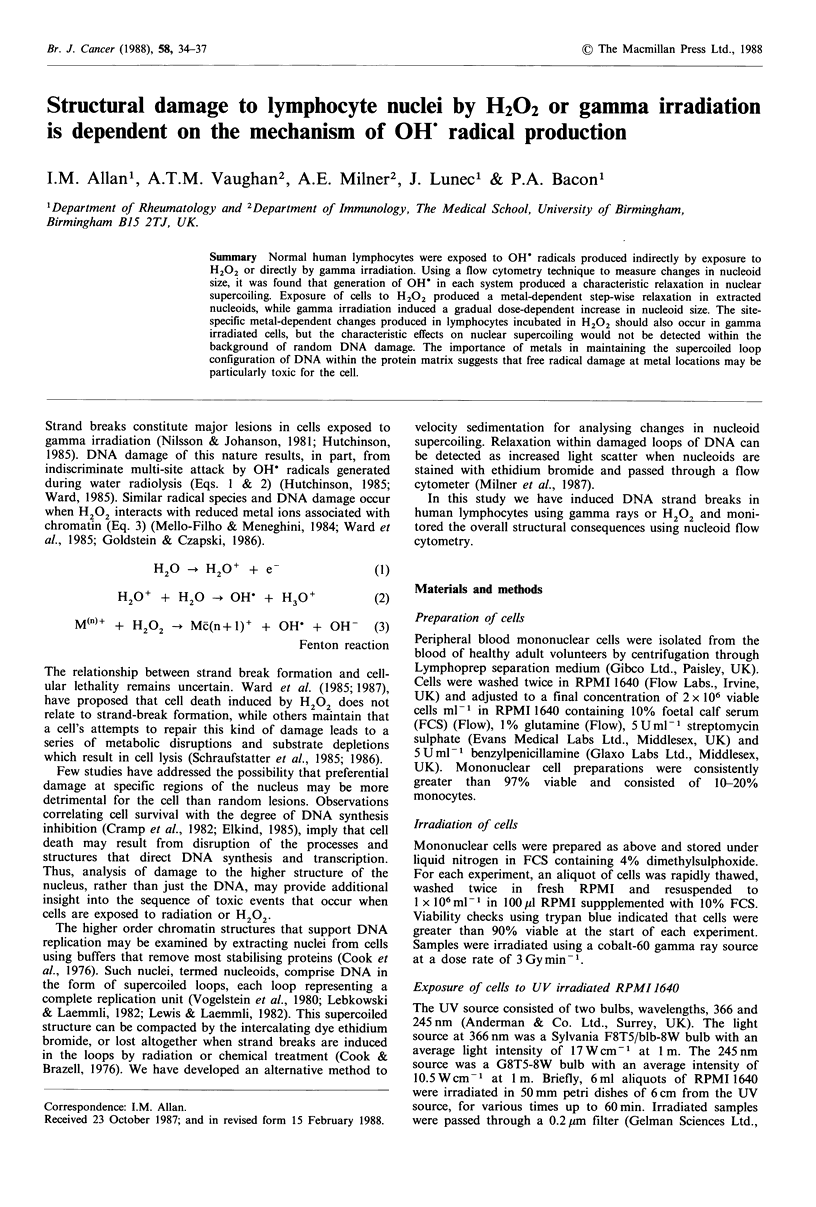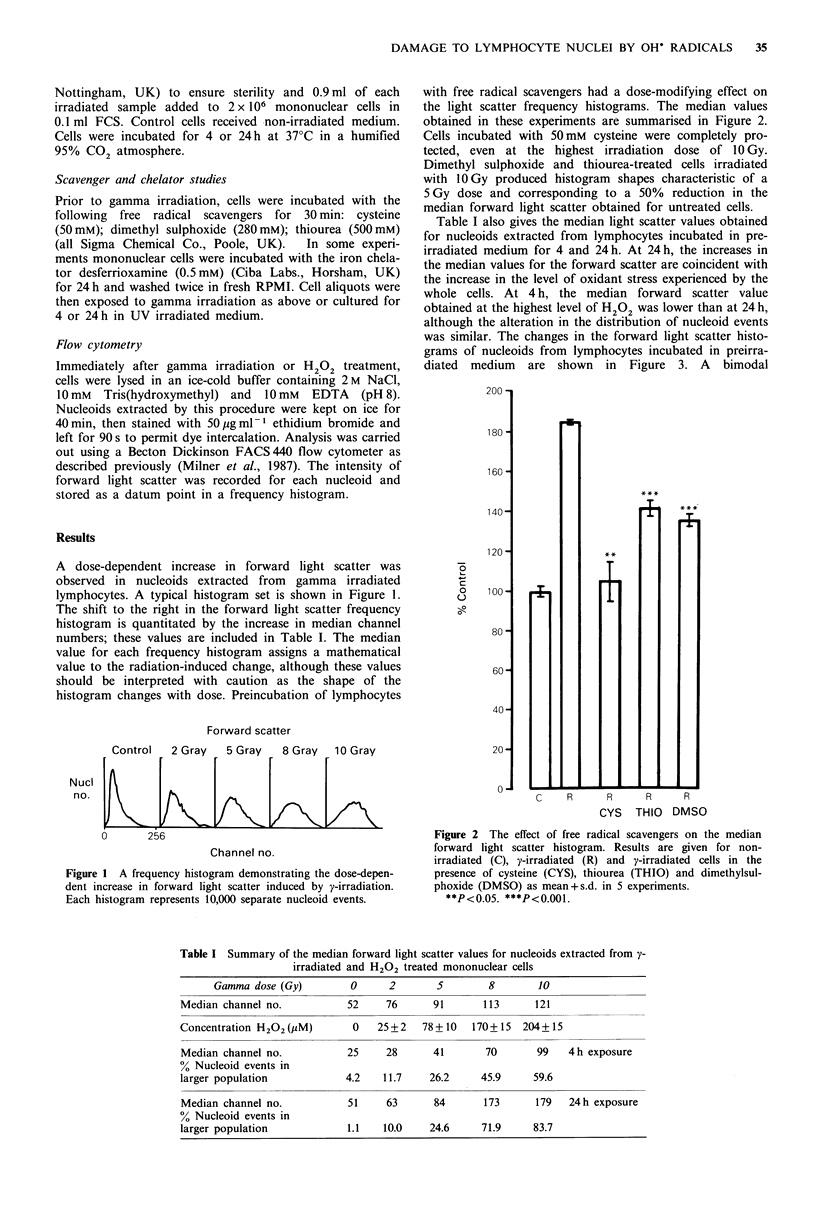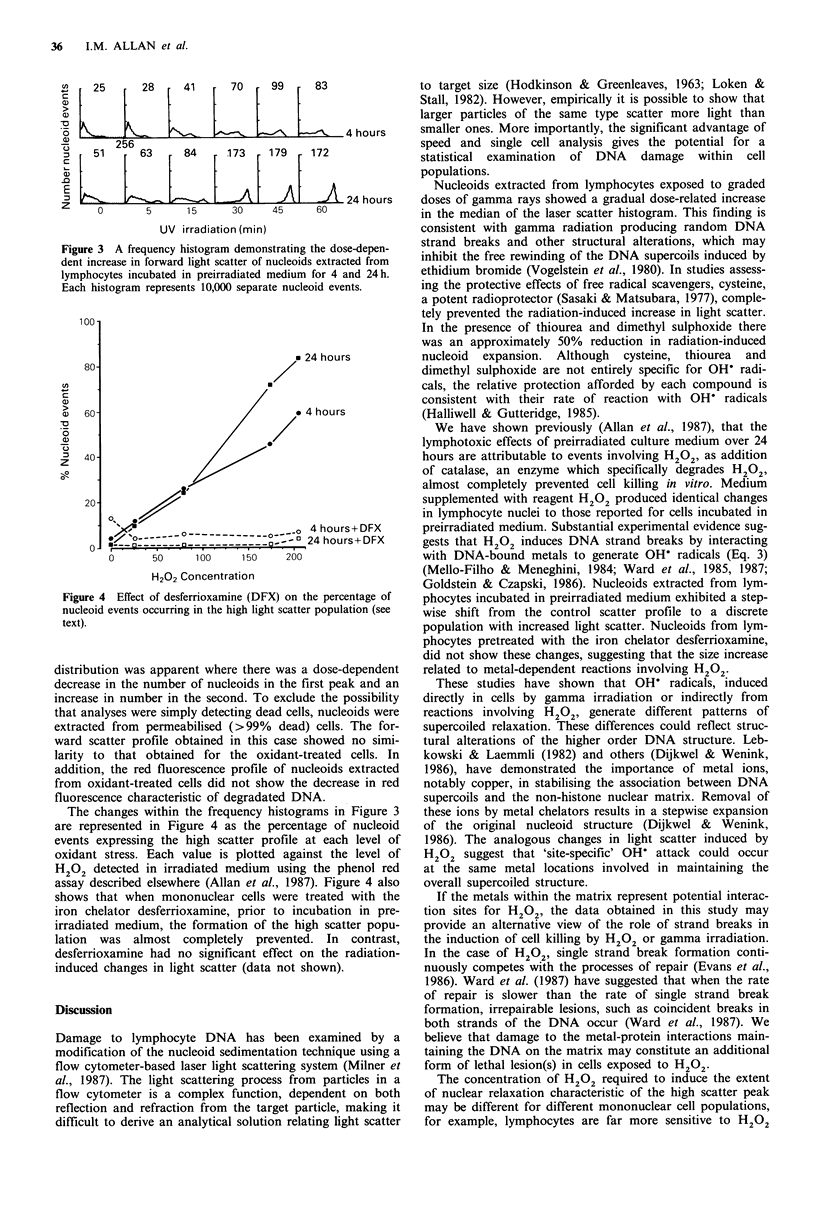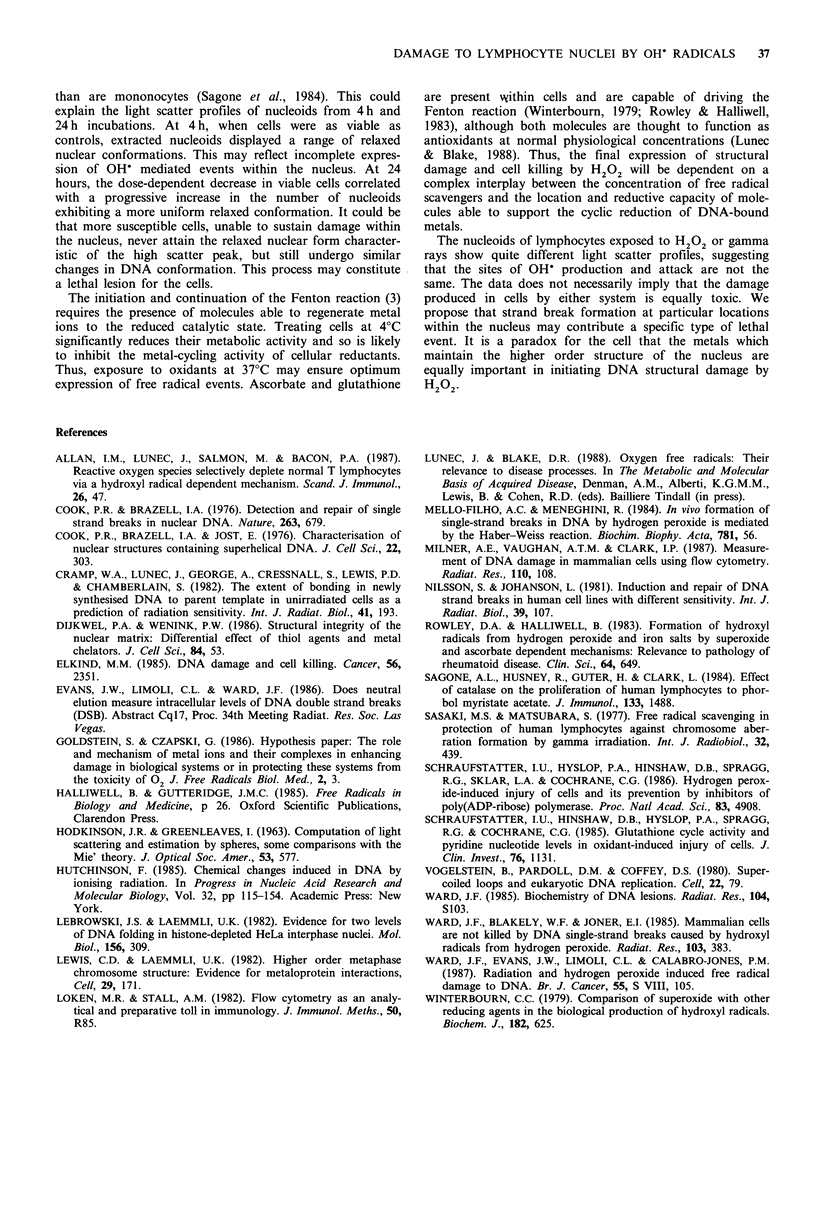# Structural damage to lymphocyte nuclei by H2O2 or gamma irradiation is dependent on the mechanism of OH. radical production.

**DOI:** 10.1038/bjc.1988.156

**Published:** 1988-07

**Authors:** I. M. Allan, A. T. Vaughan, A. E. Milner, J. Lunec, P. A. Bacon

**Affiliations:** Department of Rheumatology, Medical School, University of Birmingham, UK.

## Abstract

Normal human lymphocytes were exposed to OH. radicals produced indirectly by exposure to H2O2 or directly by gamma irradiation. Using a flow cytometry technique to measure changes in nucleoid size, it was found that generation of OH. in each system produced a characteristic relaxation in nuclear supercoiling. Exposure of cells to H2O2 produced a metal-dependent step-wise relaxation in extracted nucleoids, while gamma irradiation induced a gradual dose-dependent increase in nucleoid size. The site-specific metal-dependent changes produced in lymphocytes incubated in H2O2 should also occur in gamma irradiated cells, but the characteristic effects on nuclear supercoiling would not be detected within the background of random DNA damage. The importance of metals in maintaining the supercoiled loop configuration of DNA within the protein matrix suggests that free radical damage at metal locations may be particularly toxic for the cell.


Br. J. Cancer (1988), 58, 34–37 © The Macmillan Press Ltd., 1988

### Structural damage to lymphocyte nuclei by $H_2O_2$ or gamma irradiation is dependent on the mechanism of $OH^{\bullet}$ radical production

I.M. Allan[1], A.T.M. Vaughan[2], A.E. Milner[2], J. Lunec[1] & P.A. Bacon[1]

[1]*Department of Rheumatology and* [2]*Department of Immunology, The Medical School, University of Birmingham, Birmingham B15 2TJ, UK.*

**Summary** Normal human lymphocytes were exposed to $OH^{\bullet}$ radicals produced indirectly by exposure to $H_2O_2$ or directly by gamma irradiation. Using a flow cytometry technique to measure changes in nucleoid size, it was found that generation of $OH^{\bullet}$ in each system produced a characteristic relaxation in nuclear supercoiling. Exposure of cells to $H_2O_2$ produced a metal-dependent step-wise relaxation in extracted nucleoids, while gamma irradiation induced a gradual dose-dependent increase in nucleoid size. The site-specific metal-dependent changes produced in lymphocytes incubated in $H_2O_2$ should also occur in gamma irradiated cells, but the characteristic effects on nuclear supercoiling would not be detected within the background of random DNA damage. The importance of metals in maintaining the supercoiled loop configuration of DNA within the protein matrix suggests that free radical damage at metal locations may be particularly toxic for the cell.

Strand breaks constitute major lesions in cells exposed to gamma irradiation (Nilsson & Johanson, 1981; Hutchinson, 1985). DNA damage of this nature results, in part, from indiscriminate multi-site attack by $OH^{\bullet}$ radicals generated during water radiolysis (Eqs. 1 & 2) (Hutchinson, 1985; Ward, 1985). Similar radical species and DNA damage occur when $H_2O_2$ interacts with reduced metal ions associated with chromatin (Eq. 3) (Mello-Filho & Meneghini, 1984; Ward *et al.*, 1985; Goldstein & Czapski, 1986).

$$H_2O \rightarrow H_2O^+ + e^- \quad (1)$$

$$H_2O^+ + H_2O \rightarrow OH^{\bullet} + H_3O^+ \quad (2)$$

$$M^{(n)+} + H_2O_2 \rightarrow M\bar{c}(n+1)^+ + OH^{\bullet} + OH^- \quad (3)$$

Fenton reaction

The relationship between strand break formation and cellular lethality remains uncertain. Ward *et al.* (1985; 1987), have proposed that cell death induced by $H_2O_2$ does not relate to strand-break formation, while others maintain that a cell's attempts to repair this kind of damage leads to a series of metabolic disruptions and substrate depletions which result in cell lysis (Schraufstatter *et al.*, 1985; 1986).

Few studies have addressed the possibility that preferential damage at specific regions of the nucleus may be more detrimental for the cell than random lesions. Observations correlating cell survival with the degree of DNA synthesis inhibition (Cramp *et al.*, 1982; Elkind, 1985), imply that cell death may result from disruption of the processes and structures that direct DNA synthesis and transcription. Thus, analysis of damage to the higher structure of the nucleus, rather than just the DNA, may provide additional insight into the sequence of toxic events that occur when cells are exposed to radiation or $H_2O_2$.

The higher order chromatin structures that support DNA replication may be examined by extracting nuclei from cells using buffers that remove most stabilising proteins (Cook *et al.*, 1976). Such nuclei, termed nucleoids, comprise DNA in the form of supercoiled loops, each loop representing a complete replication unit (Vogelstein *et al.*, 1980; Lebkowski & Laemmli, 1982; Lewis & Laemmli, 1982). This supercoiled structure can be compacted by the intercalating dye ethidium bromide, or lost altogether when strand breaks are induced in the loops by radiation or chemical treatment (Cook & Brazell, 1976). We have developed an alternative method to velocity sedimentation for analysing changes in nucleoid supercoiling. Relaxation within damaged loops of DNA can be detected as increased light scatter when nucleoids are stained with ethidium bromide and passed through a flow cytometer (Milner *et al.*, 1987).

In this study we have induced DNA strand breaks in human lymphocytes using gamma rays or $H_2O_2$ and monitored the overall structural consequences using nucleoid flow cytometry.

Correspondence: I.M. Allan.
Received 23 October 1987; and in revised form 15 February 1988.

### Materials and methods

### *Preparation of cells*

Peripheral blood mononuclear cells were isolated from the blood of healthy adult volunteers by centrifugation through Lymphoprep separation medium (Gibco Ltd., Paisley, UK). Cells were washed twice in RPMI 1640 (Flow Labs., Irvine, UK) and adjusted to a final concentration of $2\times10^6$ viable cells $ml^{-1}$ in RPMI 1640 containing 10% foetal calf serum (FCS) (Flow), 1% glutamine (Flow), $5\,U\,ml^{-1}$ streptomycin sulphate (Evans Medical Labs Ltd., Middlesex, UK) and $5\,U\,ml^{-1}$ benzylpenicillamine (Glaxo Labs Ltd., Middlesex, UK). Mononuclear cell preparations were consistently greater than 97% viable and consisted of 10–20% monocytes.

### *Irradiation of cells*

Mononuclear cells were prepared as above and stored under liquid nitrogen in FCS containing 4% dimethylsulphoxide. For each experiment, an aliquot of cells was rapidly thawed, washed twice in fresh RPMI and resuspended to $1\times10^6\,ml^{-1}$ in 100 $\mu$l RPMI suppplemented with 10% FCS. Viability checks using trypan blue indicated that cells were greater than 90% viable at the start of each experiment. Samples were irradiated using a cobalt-60 gamma ray source at a dose rate of $3\,Gy\,min^{-1}$.

### *Exposure of cells to UV irradiated RPMI 1640*

The UV source consisted of two bulbs, wavelengths, 366 and 245 nm (Anderman & Co. Ltd., Surrey, UK). The light source at 366 nm was a Sylvania F8T5/blb-8W bulb with an average light intensity of $17\,W\,cm^{-1}$ at 1 m. The 245 nm source was a G8T5-8W bulb with an average intensity of $10.5\,W\,cm^{-1}$ at 1 m. Briefly, 6 ml aliquots of RPMI 1640 were irradiated in 50 mm petri dishes of 6 cm from the UV source, for various times up to 60 min. Irradiated samples were passed through a 0.2 $\mu$m filter (Gelman Sciences Ltd.,

Nottingham, UK) to ensure sterility and 0.9 ml of each irradiated sample added to $2 \times 10^6$ mononuclear cells in 0.1 ml FCS. Control cells received non-irradiated medium. Cells were incubated for 4 or 24 h at 37°C in a humified 95% $CO_2$ atmosphere.

*Scavenger and chelator studies*

Prior to gamma irradiation, cells were incubated with the following free radical scavengers for 30 min: cysteine (50 mM); dimethyl sulphoxide (280 mM); thiourea (500 mM) (all Sigma Chemical Co., Poole, UK). In some experiments mononuclear cells were incubated with the iron chelator desferrioxamine (0.5 mM) (Ciba Labs., Horsham, UK) for 24 h and washed twice in fresh RPMI. Cell aliquots were then exposed to gamma irradiation as above or cultured for 4 or 24 h in UV irradiated medium.

*Flow cytometry*

Immediately after gamma irradiation or $H_2O_2$ treatment, cells were lysed in an ice-cold buffer containing 2 M NaCl, 10 mM Tris(hydroxymethyl) and 10 mM EDTA (pH 8). Nucleoids extracted by this procedure were kept on ice for 40 min, then stained with 50 $\mu$g ml$^{-1}$ ethidium bromide and left for 90 s to permit dye intercalation. Analysis was carried out using a Becton Dickinson FACS 440 flow cytometer as described previously (Milner *et al.*, 1987). The intensity of forward light scatter was recorded for each nucleoid and stored as a datum point in a frequency histogram.

### Results

A dose-dependent increase in forward light scatter was observed in nucleoids extracted from gamma irradiated lymphocytes. A typical histogram set is shown in Figure 1. The shift to the right in the forward light scatter frequency histogram is quantitated by the increase in median channel numbers; these values are included in Table I. The median value for each frequency histogram assigns a mathematical value to the radiation-induced change, although these values should be interpreted with caution as the shape of the histogram changes with dose. Preincubation of lymphocytes with free radical scavengers had a dose-modifying effect on the light scatter frequency histograms. The median values obtained in these experiments are summarised in Figure 2. Cells incubated with 50 mM cysteine were completely protected, even at the highest irradiation dose of 10 Gy. Dimethyl sulphoxide and thiourea-treated cells irradiated with 10 Gy produced histogram shapes characteristic of a 5 Gy dose and corresponding to a 50% reduction in the median forward light scatter obtained for untreated cells.

Table I also gives the median light scatter values obtained for nucleoids extracted from lymphocytes incubated in preirradiated medium for 4 and 24 h. At 24 h, the increases in the median values for the forward scatter are coincident with the increase in the level of oxidant stress experienced by the whole cells. At 4 h, the median forward scatter value obtained at the highest level of $H_2O_2$ was lower than at 24 h, although the alteration in the distribution of nucleoid events was similar. The changes in the forward light scatter histograms of nucleoids from lymphocytes incubated in preirradiated medium are shown in Figure 3. A bimodal

**Figure 1** A frequency histogram demonstrating the dose-dependent increase in forward light scatter induced by $\gamma$-irradiation. Each histogram represents 10,000 separate nucleoid events.

**Figure 2** The effect of free radical scavengers on the median forward light scatter histogram. Results are given for non-irradiated (C), $\gamma$-irradiated (R) and $\gamma$-irradiated cells in the presence of cysteine (CYS), thiourea (THIO) and dimethylsulphoxide (DMSO) as mean+s.d. in 5 experiments. $^{**}P<0.05$. $^{***}P<0.001$.

**Table I** Summary of the median forward light scatter values for nucleoids extracted from $\gamma$-irradiated and $H_2O_2$ treated mononuclear cells

| *Gamma dose (Gy)* | *0* | *2* | *5* | *8* | *10* | |
|---|---|---|---|---|---|---|
| Median channel no. | 52 | 76 | 91 | 113 | 121 | |
| Concentration $H_2O_2$ ($\mu$M) | 0 | 25±2 | 78±10 | 170±15 | 204±15 | |
| Median channel no. | 25 | 28 | 41 | 70 | 99 | 4 h exposure |
| % Nucleoid events in larger population | 4.2 | 11.7 | 26.2 | 45.9 | 59.6 | |
| Median channel no. | 51 | 63 | 84 | 173 | 179 | 24 h exposure |
| % Nucleoid events in larger population | 1.1 | 10.0 | 24.6 | 71.9 | 83.7 | |

**Figure 3** A frequency histogram demonstrating the dose-dependent increase in forward light scatter of nucleoids extracted from lymphocytes incubated in preirradiated medium for 4 and 24 h. Each histogram represents 10,000 separate nucleoid events.

**Figure 4** Effect of desferrioxamine (DFX) on the percentage of nucleoid events occurring in the high light scatter population (see text).

distribution was apparent where there was a dose-dependent decrease in the number of nucleoids in the first peak and an increase in number in the second. To exclude the possibility that analyses were simply detecting dead cells, nucleoids were extracted from permeabilised (>99% dead) cells. The forward scatter profile obtained in this case showed no similarity to that obtained for the oxidant-treated cells. In addition, the red fluorescence profile of nucleoids extracted from oxidant-treated cells did not show the decrease in red fluorescence characteristic of degradated DNA.

The changes within the frequency histograms in Figure 3 are represented in Figure 4 as the percentage of nucleoid events expressing the high scatter profile at each level of oxidant stress. Each value is plotted against the level of $H_2O_2$ detected in irradiated medium using the phenol red assay described elsewhere (Allan *et al.*, 1987). Figure 4 also shows that when mononuclear cells were treated with the iron chelator desferrioxamine, prior to incubation in pre-irradiated medium, the formation of the high scatter population was almost completely prevented. In contrast, desferrioxamine had no significant effect on the radiation-induced changes in light scatter (data not shown).

### Discussion

Damage to lymphocyte DNA has been examined by a modification of the nucleoid sedimentation technique using a flow cytometer-based laser light scattering system (Milner *et al.*, 1987). The light scattering process from particles in a flow cytometer is a complex function, dependent on both reflection and refraction from the target particle, making it difficult to derive an analytical solution relating light scatter to target size (Hodkinson & Greenleaves, 1963; Loken & Stall, 1982). However, empirically it is possible to show that larger particles of the same type scatter more light than smaller ones. More importantly, the significant advantage of speed and single cell analysis gives the potential for a statistical examination of DNA damage within cell populations.

Nucleoids extracted from lymphocytes exposed to graded doses of gamma rays showed a gradual dose-related increase in the median of the laser scatter histogram. This finding is consistent with gamma radiation producing random DNA strand breaks and other structural alterations, which may inhibit the free rewinding of the DNA supercoils induced by ethidium bromide (Vogelstein *et al.*, 1980). In studies assessing the protective effects of free radical scavengers, cysteine, a potent radioprotector (Sasaki & Matsubara, 1977), completely prevented the radiation-induced increase in light scatter. In the presence of thiourea and dimethyl sulphoxide there was an approximately 50% reduction in radiation-induced nucleoid expansion. Although cysteine, thiourea and dimethyl sulphoxide are not entirely specific for OH$^\bullet$ radicals, the relative protection afforded by each compound is consistent with their rate of reaction with OH$^\bullet$ radicals (Halliwell & Gutteridge, 1985).

We have shown previously (Allan *et al.*, 1987), that the lymphotoxic effects of preirradiated culture medium over 24 hours are attributable to events involving $H_2O_2$, as addition of catalase, an enzyme which specifically degrades $H_2O_2$, almost completely prevented cell killing *in vitro*. Medium supplemented with reagent $H_2O_2$ produced identical changes in lymphocyte nuclei to those reported for cells incubated in preirradiated medium. Substantial experimental evidence suggests that $H_2O_2$ induces DNA strand breaks by interacting with DNA-bound metals to generate OH$^\bullet$ radicals (Eq. 3) (Mello-Filho & Meneghini, 1984; Ward *et al.*, 1985, 1987; Goldstein & Czapski, 1986). Nucleoids extracted from lymphocytes incubated in preirradiated medium exhibited a stepwise shift from the control scatter profile to a discrete population with increased light scatter. Nucleoids from lymphocytes pretreated with the iron chelator desferrioxamine, did not show these changes, suggesting that the size increase related to metal-dependent reactions involving $H_2O_2$.

These studies have shown that OH$^\bullet$ radicals, induced directly in cells by gamma irradiation or indirectly from reactions involving $H_2O_2$, generate different patterns of supercoiled relaxation. These differences could reflect structural alterations of the higher order DNA structure. Lebkowski & Laemmli (1982) and others (Dijkwel & Wenink, 1986), have demonstrated the importance of metal ions, notably copper, in stabilising the association between DNA supercoils and the non-histone nuclear matrix. Removal of these ions by metal chelators results in a stepwise expansion of the original nucleoid structure (Dijkwel & Wenink, 1986). The analogous changes in light scatter induced by $H_2O_2$ suggest that 'site-specific' OH$^\bullet$ attack could occur at the same metal locations involved in maintaining the overall supercoiled structure.

If the metals within the matrix represent potential interaction sites for $H_2O_2$, the data obtained in this study may provide an alternative view of the role of strand breaks in the induction of cell killing by $H_2O_2$ or gamma irradiation. In the case of $H_2O_2$, single strand break formation continuously competes with the processes of repair (Evans *et al.*, 1986). Ward *et al.* (1987) have suggested that when the rate of repair is slower than the rate of single strand break formation, irrepairable lesions, such as coincident breaks in both strands of the DNA occur (Ward *et al.*, 1987). We believe that damage to the metal-protein interactions maintaining the DNA on the matrix may constitute an additional form of lethal lesion(s) in cells exposed to $H_2O_2$.

The concentration of $H_2O_2$ required to induce the extent of nuclear relaxation characteristic of the high scatter peak may be different for different mononuclear cell populations, for example, lymphocytes are far more sensitive to $H_2O_2$

than are mononocytes (Sagone *et al.*, 1984). This could explain the light scatter profiles of nucleoids from 4 h and 24 h incubations. At 4 h, when cells were as viable as controls, extracted nucleoids displayed a range of relaxed nuclear conformations. This may reflect incomplete expression of OH$^{\bullet}$ mediated events within the nucleus. At 24 hours, the dose-dependent decrease in viable cells correlated with a progressive increase in the number of nucleoids exhibiting a more uniform relaxed conformation. It could be that more susceptible cells, unable to sustain damage within the nucleus, never attain the relaxed nuclear form characteristic of the high scatter peak, but still undergo similar changes in DNA conformation. This process may constitute a lethal lesion for the cells.

The initiation and continuation of the Fenton reaction (3) requires the presence of molecules able to regenerate metal ions to the reduced catalytic state. Treating cells at 4°C significantly reduces their metabolic activity and so is likely to inhibit the metal-cycling activity of cellular reductants. Thus, exposure to oxidants at 37°C may ensure optimum expression of free radical events. Ascorbate and glutathione are present within cells and are capable of driving the Fenton reaction (Winterbourn, 1979; Rowley & Halliwell, 1983), although both molecules are thought to function as antioxidants at normal physiological concentrations (Lunec & Blake, 1988). Thus, the final expression of structural damage and cell killing by $H_2O_2$ will be dependent on a complex interplay between the concentration of free radical scavengers and the location and reductive capacity of molecules able to support the cyclic reduction of DNA-bound metals.

The nucleoids of lymphocytes exposed to $H_2O_2$ or gamma rays show quite different light scatter profiles, suggesting that the sites of OH$^{\bullet}$ production and attack are not the same. The data does not necessarily imply that the damage produced in cells by either system is equally toxic. We propose that strand break formation at particular locations within the nucleus may contribute a specific type of lethal event. It is a paradox for the cell that the metals which maintain the higher order structure of the nucleus are equally important in initiating DNA structural damage by $H_2O_2$.

### References

ALLAN, I.M., LUNEC, J., SALMON, M. & BACON, P.A. (1987). Reactive oxygen species selectively deplete normal T lymphocytes via a hydroxyl radical dependent mechanism. *Scand. J. Immunol.*, **26**, 47.

COOK, P.R. & BRAZELL, I.A. (1976). Detection and repair of single strand breaks in nuclear DNA. *Nature*, **263**, 679.

COOK, P.R., BRAZELL, I.A. & JOST, E. (1976). Characterisation of nuclear structures containing superhelical DNA. *J. Cell Sci.*, **22**, 303.

CRAMP, W.A., LUNEC, J., GEORGE, A., CRESSNALL, S., LEWIS, P.D. & CHAMBERLAIN, S. (1982). The extent of bonding in newly synthesised DNA to parent template in unirradiated cells as a prediction of radiation sensitivity. *Int. J. Radiat. Biol.*, **41**, 193.

DIJKWEL, P.A. & WENINK, P.W. (1986). Structural integrity of the nuclear matrix: Differential effect of thiol agents and metal chelators. *J. Cell Sci.*, **84**, 53.

ELKIND, M.M. (1985). DNA damage and cell killing. *Cancer*, **56**, 2351.

EVANS, J.W., LIMOLI, C.L. & WARD, J.F. (1986). Does neutral elution measure intracellular levels of DNA double strand breaks (DSB). Abstract Cq17, Proc. 34th Meeting Radiat. *Res. Soc. Las Vegas.*

GOLDSTEIN, S. & CZAPSKI, G. (1986). Hypothesis paper: The role and mechanism of metal ions and their complexes in enhancing damage in biological systems or in protecting these systems from the toxicity of $O_2$ *J. Free Radicals Biol. Med.*, **2**, 3.

HALLIWELL, B. & GUTTERIDGE, J.M.C. (1985). *Free Radicals in Biology and Medicine*, p 26. Oxford Scientific Publications, Clarendon Press.

HODKINSON, J.R. & GREENLEAVES, I. (1963). Computation of light scattering and estimation by spheres, some comparisons with the Mie' theory. *J. Optical Soc. Amer.*, **53**, 577.

HUTCHINSON, F. (1985). Chemical changes induced in DNA by ionising radiation. In *Progress in Nucleic Acid Research and Molecular Biology*, Vol. 32, pp 115–154. Academic Press: New York.

LEBROWSKI, J.S. & LAEMMLI, U.K. (1982). Evidence for two levels of DNA folding in histone-depleted HeLa interphase nuclei. *Mol. Biol.*, **156**, 309.

LEWIS, C.D. & LAEMMLI, U.K. (1982). Higher order metaphase chromosome structure: Evidence for metaloprotein interactions, *Cell*, **29**, 171.

LOKEN, M.R. & STALL, A.M. (1982). Flow cytometry as an analytical and preparative toll in immunology. *J. Immunol. Meths.*, **50**, R85.

LUNEC, J. & BLAKE, D.R. (1988). Oxygen free radicals: Their relevance to disease processes. In *The Metabolic and Molecular Basis of Acquired Disease*, Denman, A.M., Alberti, K.G.M.M., Lewis, B. & Cohen, R.D. (eds). Bailliere Tindall (in press).

MELLO-FILHO, A.C. & MENEGHINI, R. (1984). *In vivo* formation of single-strand breaks in DNA by hydrogen peroxide is mediated by the Haber–Weiss reaction. *Biochim. Biophy. Acta*, **781**, 56.

MILNER, A.E., VAUGHAN, A.T.M. & CLARK, I.P. (1987). Measurement of DNA damage in mammalian cells using flow cytometry. *Radiat. Res.*, **110**, 108.

NILSSON, S. & JOHANSON, L. (1981). Induction and repair of DNA strand breaks in human cell lines with different sensitivity. *Int. J. Radiat. Biol.*, **39**, 107.

ROWLEY, D.A. & HALLIWELL, B. (1983). Formation of hydroxyl radicals from hydrogen peroxide and iron salts by superoxide and ascorbate dependent mechanisms: Relevance to pathology of rheumatoid disease. *Clin. Sci.*, **64**, 649.

SAGONE, A.L., HUSNEY, R., GUTER, H. & CLARK, L. (1984). Effect of catalase on the proliferation of human lymphocytes to phorbol myristate acetate. *J. Immunol.*, **133**, 1488.

SASAKI, M.S. & MATSUBARA, S. (1977). Free radical scavenging in protection of human lymphocytes against chromosome aberration formation by gamma irradiation. *Int. J. Radiobiol.*, **32**, 439.

SCHRAUFSTATTER, I.U., HYSLOP, P.A., HINSHAW, D.B., SPRAGG, R.G., SKLAR, L.A. & COCHRANE, C.G. (1986). Hydrogen peroxide-induced injury of cells and its prevention by inhibitors of poly(ADP-ribose) polymerase. *Proc. Natl Acad. Sci.*, **83**, 4908.

SCHRAUFSTATTER, I.U., HINSHAW, D.B., HYSLOP, P.A., SPRAGG, R.G. & COCHRANE, C.G. (1985). Glutathione cycle activity and pyridine nucleotide levels in oxidant-induced injury of cells. *J. Clin. Invest.*, **76**, 1131.

VOGELSTEIN, B., PARDOLL, D.M. & COFFEY, D.S. (1980). Supercoiled loops and eukaryotic DNA replication. *Cell*, **22**, 79.

WARD, J.F. (1985). Biochemistry of DNA lesions. *Radiat. Res.*, **104**, S103.

WARD, J.F., BLAKELY, W.F. & JONER, E.I. (1985). Mammalian cells are not killed by DNA single-strand breaks caused by hydroxyl radicals from hydrogen peroxide. *Radiat. Res.*, **103**, 383.

WARD, J.F., EVANS, J.W., LIMOLI, C.L. & CALABRO-JONES, P.M. (1987). Radiation and hydrogen peroxide induced free radical damage to DNA. *Br. J. Cancer*, **55**, S VIII, 105.

WINTERBOURN, C.C. (1979). Comparison of superoxide with other reducing agents in the biological production of hydroxyl radicals. *Biochem. J.*, **182**, 625.